# Development and Evaluation of Mesoporous SiO_2_ Nanoparticle-Based Sustained-Release Gel Breaker for Clean Fracturing Fluids

**DOI:** 10.3390/polym17152078

**Published:** 2025-07-30

**Authors:** Guiqiang Fei, Banghua Liu, Liyuan Guo, Yuan Chang, Boliang Xue

**Affiliations:** College of Chemistry and Chemical Engineering, Shaanxi University of Science & Technology, Xi’an 710021, China; feiguiqiang@sust.edu.cn (G.F.); guoliyuan@sust.edu.cn (L.G.); 240812097@sust.edu.cn (Y.C.); 240811086@sust.edu.cn (B.X.)

**Keywords:** coalbed methane, fracturing fluid, mesoporous silica, controlled-release breaker, paraffin coating

## Abstract

To address critical technical challenges in coalbed methane fracturing, including the uncontrollable release rate of conventional breaker agents and incomplete gel breaking, this study designs and fabricates an intelligent controlled-release breaker system based on paraffin-coated mesoporous silica nanoparticle carriers. Three types of mesoporous silica (MSN) carriers with distinct pore sizes are synthesized via the sol-gel method using CTAB, P123, and F127 as structure-directing agents, respectively. Following hydrophobic modification with octyltriethoxysilane, n-butanol breaker agents are loaded into the carriers, and a temperature-responsive controlled-release system is constructed via paraffin coating technology. The pore size distribution was analyzed by the BJH model, confirming that the average pore diameters of CTAB-MSNs, P123-MSNs, and F127-MSNs were 5.18 nm, 6.36 nm, and 6.40 nm, respectively. The BET specific surface areas were 686.08, 853.17, and 946.89 m^2^/g, exhibiting an increasing trend with the increase in pore size. Drug-loading performance studies reveal that at the optimal loading concentration of 30 mg/mL, the loading efficiencies of n-butanol on the three carriers reach 28.6%, 35.2%, and 38.9%, respectively. The release behavior study under simulated reservoir temperature conditions (85 °C) reveals that the paraffin-coated system exhibits a distinct three-stage release pattern: a lag phase (0–1 h) caused by paraffin encapsulation, a rapid release phase (1–8 h) induced by high-temperature concentration diffusion, and a sustained release phase (8–30 h) attributed to nano-mesoporous characteristics. This intelligent controlled-release breaker demonstrates excellent temporal compatibility with coalbed methane fracturing processes, providing a novel technical solution for the efficient and clean development of coalbed methane.

## 1. Introduction

As a clean energy resource, coalbed methane (CBM) development faces the dual challenges of low reservoir permeability and strong adsorption characteristics [1,2]. In CBM exploitation, hydraulic fracturing has become a key technology to enhance gas production, with efficient fracturing fluid systems being critical for forming effective fracture networks [3,4]. Liu et al. [5] demonstrated that the rational design of fracturing fluid systems is decisive for the success of CBM development. Previous studies have successfully constructed a clean fracturing fluid system based on ultra-long chain gemini surfactants, which exhibit an excellent proppant-carrying capacity and low residue characteristics [6,7]. However, traditional breakers used in this system suffer from three major limitations: uncontrollable release rates leading to the premature loss of proppant-carrying capacity or incomplete gel breaking; insufficient gel breaking causing substantial residue accumulation in reservoirs; and poor compatibility with coal rocks, which compromises post-fracturing productivity [8].

In recent years, nanomaterials have gained increasing applications in oil and gas development [9,10]. Yang et al. [11] highlighted the unique advantages of nanomaterials in petroleum chemistry, particularly as controlled-release carriers. Mesoporous silica nanoparticles (MSNs), with their tunable pore structures, large specific surface areas, and good biocompatibility, have emerged as ideal drug carriers and sustained-release materials [12,13]. Zhao et al. [14] provided experimental evidence for the significant cargo-loading capability and sustained-release properties of mesoporous silica nanoparticles (MSNs) in their role as delivery carriers. Utilizing MSNs as breaker carriers holds promise for addressing the limitations of traditional breakers by enabling controlled release and efficient gel breaking. Recent studies by Fan et al. [15] developed a temperature-responsive controlled-release system for fracturing fluids based on MSNs, demonstrating the significant effects of different pore structures on the release kinetics of active components. Zhong et al. [16] further functionalized MSN surfaces to achieve multi-stimuli-responsive controlled release, providing new insights for the targeted delivery of oilfield chemicals. Additionally, Kabiri et al. [17] conducted hydrophobic modification on the surface of mesoporous silica nanoparticles (MSNs), which effectively inhibited metal ion-induced aggregation in high-salinity environments and improved the loading efficiency of hydrophobic molecules. This study is of great significance for applications in complex formation conditions.

To address the key technical issues of difficult regulation of release rate and incomplete gel breaking by traditional gel breakers in coalbed methane fracturing, this study designs an intelligent controlled-release gel breaker system with paraffin-coated mesoporous silica nanocarriers. Through the regulation of carrier pore structure and temperature-responsive coating technology, the controlled release of gel breakers and efficient gel breaking are achieved. The influence rules of carrier structure on loading performance and release behavior are systematically studied, the structure–activity relationship between pore structure parameters and gel breaking performance is established, and the gel-breaking effect and environmental compatibility are comprehensively evaluated, providing a new technical solution for the efficient and clean development of coalbed methane.

## 2. Experimental Methodology

### 2.1. Materials and Equipment

Tetraethoxysilane (TEOS, analytical grade); cetyltrimethylammonium bromide (CTAB, analytical grade); triblock copolymer Pluronic P123 (average molecular weight Mw ≈ 5800); triblock copolymer Pluronic F127 (average molecular weight Mw ≈ 12,600); concentrated hydrochloric acid (37% mass fraction); ammonia water (25% mass fraction); 1,3,5-trimethylbenzene (TMB, purity 98%); ammonium persulfate (APS, analytical grade); n-butanol (analytical grade); absolute ethanol (analytical grade); sodium hydroxide (analytical grade); ultra-long chain cationic surfactant (laboratory-synthesized, confirmed by ^1^H nuclear magnetic resonance and infrared spectroscopy, purity > 98%); deionized water (prepared by a laboratory Millipore ultrapure water system, resistivity ≥ 18.2 MΩ·cm). Coal sample characterization: The coal sample used in this study was obtained from a CBM field in Shanxi Province, China. Proximate analysis (dry basis): volatile matter 28.5%, ash content 12.3%, and fixed carbon 59.2%. Ultimate analysis (dry ash-free basis): C 85.2%, H 4.8%, N 1.2%, S 0.6%, and O 8.2%. The coal rank is classified as high volatile bituminous coal with vitrinite reflectance (Ro) of 0.78%. All commercially purchased chemical reagents were used without further purification.

The experimental instruments used are shown in Table 1:

### 2.2. Preparation of Paraffin-Encapsulated n-Butanol-Loaded Mesoporous Silica Gel Breaker

**Figure 1:**
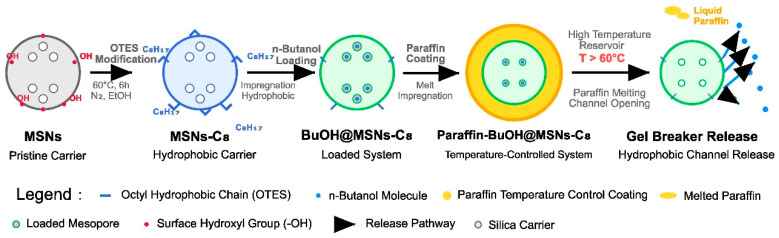
Technical Roadmap for the Preparation of Paraffin-Wrapped Mesoporous Silica-Supported n-Butanol Gel Breaker.

The sustained release and delayed release of breakers are key technologies to achieve precise gel breaking of VES fracturing fluid. In this study, mesoporous silica was employed as the carrier material to realize the effective loading of a n-butanol breaker through its high specific surface area and tunable pore structure. To prevent the premature release of the breaker during the fracturing and fracture creation stage, a temperature-controlled delayed release system was further constructed via paraffin wax coating technology. When the fracturing fluid enters the high-temperature reservoir environment, the melting of paraffin wax triggers the release of the breaker. Combined with the sustained release property of the mesoporous carrier, precise control of the gel breaking time is achieved. The preparation process is shown in Figure 1.

#### 2.2.1. Synthesis of Mesoporous Silica Nanoparticles (MSNs) with Diverse Pore Sizes

Three mesoporous silica materials were synthesized through the sol-gel methodology, incorporating distinct surfactant templates. For the fabrication of CTAB-MSNs, 1.0 g of cetyltrimethylammonium bromide (CTAB) was dissolved in 480 mL of deionized water at 60 °C under magnetic stirring at 500 rpm for 30 min. The solution’s pH was adjusted to 10.5 ± 0.2 using 3.5 mL of aqueous ammonia, followed by the gradual addition of 5.0 mL of tetraethyl orthosilicate (TEOS) at a rate of 0.17 mL/min. After stirring at ambient temperature for 2 h, the resultant mixture was transferred to a Teflon-lined autoclave for hydrothermal treatment at 80 °C for 24 h.

In the synthesis of P123-MSNs, 4.0 g of P123 was dissolved in a mixture of 120 mL deionized water and 30 mL concentrated hydrochloric acid at 40 °C under continuous stirring for 4 h. Subsequently, 8.5 g of TEOS was introduced, and the stirring was maintained for an additional 20 h. The resulting mixture was then aged at 100 °C for 24 h to complete the synthesis.

During the preparation of F127-MSNs, 2.0 g of F127 was dissolved in 60 mL of 2 M hydrochloric acid at ambient temperature for 2 h. Subsequently, 2.0 g of TMB (pore-expanding agent) was introduced and the mixture was stirred for 1 h, followed by the addition of 4.25 g of TEOS. The resultant solution was continuously stirred at room temperature for 24 h and, finally, subjected to static aging at 100 °C for 48 h.

All products were separated by centrifugation/filtration, thoroughly washed with deionized water and ethanol, and dried. The organic templates were completely removed by calcination at 550 °C (for CTAB-MSNs), 500 °C (for P123-MSNs), and 350 °C (for F127-MSNs) for 6–8 h, respectively.

#### 2.2.2. Mesoporous Silica-Supported n-Butanol Gel Breaker

During the fracturing of coalbed methane (CBM) reservoirs, although VES fracturing fluid can undergo partial gel breaking under the action of coalbed methane [18], the degree of gel breaking is influenced by factors such as coalbed methane concentration, reservoir permeability, and contact time. Consequently, the gel-breaking effect is often insufficient and uncontrollable. Therefore, it is necessary to further add a breaker to ensure the complete gel breaking of the fracturing fluid. For VES fracturing fluids, commonly used breakers include oxidizing breakers (e.g., ammonium persulfate, hydrogen peroxide) [19], enzymatic breakers (e.g., α-amylase, cellulase) [7], and alcohol-based breakers (e.g., methanol, isopropanol, n-butanol) [6]. In this study, n-butanol was selected as the breaker. As a polar solvent, n-butanol can disrupt the hydrogen bond network structure between VES molecules, reduce the critical packing parameter of micelles, and transform worm-like micelles into spherical micelles, thereby causing the fracturing fluid to lose its viscoelasticity.

The specific preparation procedures are as follows:

Carrier pretreatment: Place 2.0 g of dry mesoporous silica nanoparticles (MSNs) into a 100 mL three-necked flask and pre-dry them at 110 °C for 4 h to remove surface-adsorbed water. Prepare 50 mL of a 2 wt% OTES (octyltriethoxysilane) anhydrous ethanol solution, add the pre-dried MSNs into the solution, and perform surface hydrophobization modification via reflux reaction at 60 °C for 6 h under nitrogen protection. After the reaction, wash the product three times with 20 mL of anhydrous ethanol each time, and dry it at 80 °C for 4 h to obtain the hydrophobized MSN carrier (denoted as MSNs-C_8_).

n-Butanol loading process: Prepare the n-butanol loading solution by mixing n-butanol and anhydrous ethanol at a volume ratio of 1:3 to obtain 100 mL of the loading solution. Weigh 1.5 g of the hydrophobized MSN carrier, place it in a vacuum desiccator, and evacuate to −0.08 MPa for 10 min to expel the gas in the pore channels. Slowly add 60 mL of the loading solution under vacuum conditions, then gradually restore the pressure to atmospheric pressure to promote the infiltration of the solution into the pore channels via pressure difference. Transfer the system to a magnetic stirrer, stir at room temperature for 2 h, and then let it stand and soak for 12 h to ensure that n-butanol fully enters the pore channels.

Solvent removal: Rotate and evaporate the loaded suspension at 50 °C for 2 h to remove the ethanol carrier solvent. Subsequently, dry it in a vacuum oven at 60 °C for 4 h (vacuum degree: −0.06 MPa) to further remove residual solvents, yielding n-butanol-loaded MSNs (denoted as BuOH@MSNs-C_8_).

#### 2.2.3. The Paraffin Coating Process

During the fracturing process, gel breakers inevitably come into contact with formation water and fracturing fluid additives. To prevent the premature release of the gel breaker during the fracturing and fracture-creating stage, which may affect the fracturing effect, paraffin was used to coat and seal the mesoporous silica nanoparticles (MSNs) loaded with n-butanol gel breaker [11]. The melting point of paraffin is 65–70 °C. When the fracturing fluid penetrates into the formation, the paraffin gradually melts under the action of the reservoir temperature (usually 70–90 °C), thus realizing the temperature-controlled delayed release of the gel breaker [20]. Furthermore, by combining the porous characteristics and controllable pore size distribution of mesoporous silica, the sustained release of the gel breaker is achieved, ensuring that gel breaking is completed within an appropriate time window (6–12 h) after the fracturing operation, meeting the needs of subsequent flowback and production increase.

The specific preparation process is as follows:

Preparation of paraffin solution: Accurately weigh 5.0 g of paraffin, add 50 mL of n-hexane, and heat and stir in an 80 °C water bath until completely dissolved to prepare a 10 wt% paraffin-n-hexane solution. Cool to room temperature for later use.

Coating treatment process: Weigh 2.0 g of BuOH@MSNs—C_8_ and place it in a 100 mL beaker. Slowly add 30 mL of the paraffin solution and gently stir with a glass rod for 5 min to ensure complete wetting of the carrier particles. Subsequently, the reaction system was subjected to rapid cooling treatment in a −10 °C refrigerator for 30 min, prompting the paraffin to rapidly solidify on the surface of the carrier and at the orifice positions, thereby forming a sealing layer structure.

Solvent removal and post-treatment: The cooled sample was naturally volatilized of n-hexane at room temperature for 24 h, during which it was gently stirred every 6 h to prevent caking. After complete volatilization, the sample was passed through an 80-mesh sieve to remove large particle agglomerates, and the paraffin-coated n-butanol-loaded MSNs (denoted as BuOH@Paraffin-MSNs-C_8_) were obtained.

### 2.3. Methods for Material Characterization

#### 2.3.1. Fourier Transform Infrared Spectroscopy (FTIR)

An appropriate amount of dried sample was ground into powder in an agate mortar, and then uniformly mixed with pre-dried KBr at a mass ratio of 1:100. The mixture was pressed into transparent tablets with a thickness of 1–2 mm under a pressure of 8 MPa. FTIR spectroscopy measurements were performed using a Thermo Scientific Nicolet iS20 spectrometer. The testing parameters were as follows: wavenumber range of 400–4000 cm^−1^, resolution of 4 cm^−1^, 2 mg of dried sample, 13 mm tablet diameter, 32 accumulated scans, and KBr pellet as the reference.

#### 2.3.2. Analysis of Specific Surface Area and Pore Structure (BET)

The samples were first pulverized into uniform powders and subjected to vacuum degassing at 120 °C for 6 h to eliminate surface-adsorbed moisture and extraneous substances. N_2_ adsorption–desorption isotherms were subsequently recorded at liquid nitrogen temperature (77.3 K) using a Micromeritics ASAP 2460 surface area and porosimetry analyzer (USA). The calculation of specific surface area using the BET equation is based on the multi-molecular layer adsorption theory. The linear fitting method is employed to calculate the monolayer saturation adsorption capacity, thereby deriving the specific surface area of the material. The BJH model, grounded in the Kelvin equation and statistical thickness theory, utilizes nitrogen desorption isotherm data to analyze the pore size distribution and pore volume within the mesoporous range (2–50 nm). It determines the pore volume of each pore size range sequentially from high relative pressure to low pressure through a stepwise calculation approach.

#### 2.3.3. Thermogravimetric Analysis

Five to ten milligrams of sample powder were weighed and placed in an Al_2_O_3_ crucible, and thermogravimetric analysis was conducted using a Netzsch STA 2500 synchronous thermal analyzer. The test conditions were as follows: nitrogen protective atmosphere with a flow rate of 50 mL/min, a heating rate of 10 °C/min, a test temperature range of 25−800 °C, and a total test duration of 77.5 min. During the experiment, the relationship between the mass change of the sample and the temperature was continuously recorded to obtain the thermogravimetric curve (TG) and derivative thermogravimetric curve (DTG). By analyzing the weight loss steps in the TG curve, the thermal decomposition behavior of the material in different temperature intervals was determined. Combined with the number and position of peaks in the DTG curve, the number of stages and mechanism of the thermal decomposition process were judged to evaluate the thermal stability of the material.

### 2.4. Methods for Performance Evaluation

#### 2.4.1. Evaluation of Loading Performance

Weigh 100 mg of hydrophobic modified MSNs carrier, add it to 10 mL of n-butanol gel breaker solutions with different concentrations (5, 10, 20, 30, and 50 mg/mL), and carry out magnetic stirring at 25 °C for 12 h. After centrifugal separation (10,000 r/min, 15 min), the residual concentration of n-butanol in the supernatant is determined by gas chromatography. The drug loading amount of the gel breaker is calculated according to the following formula:

(1) $$DL=\frac{\left(C_{0}-C_{\varepsilon }\right)\times V}{m_{MSNs}}\times 100\%$$

In the equation: *DL* represents the loading capacity (%); *C*_0_ denotes the initial breaker concentration (mg/mL); *C*_*ε*_ is the equilibrium concentration; *V* is the solution volume (ml); and *m_MSNs_* signifies the mass of the carrier (mg). 

#### 2.4.2. Evaluation of Sustained-Release Performance

Fifty milligrams of paraffin-wrapped samples were weighed and dispersed in 50 mL of synthetic formation water, which was composed of 18.0 g/L NaCl, 3.2 g/L CaCl_2_·2H_2_O, and 1.8 g/L MgCl_2_·6H_2_O, with a total salinity of approximately 23.0 g/L. The mixture was oscillated at 150 r/min under a temperature program of 25 °C (0–30 min) → 50 °C (30 min–1 h) → 85 °C (1–30 h) to simulate the high-temperature reservoir environment underground. Pressure conditions were tested using a high-temperature and high-pressure reactor (20–30 MPa).

#### 2.4.3. Analysis of Gel Breaking Degree

The preparation process of the VES fracturing fluid base fluid with a concentration of 3.0 wt% is as follows: it consists of 3.0 wt% ultra-long chain cationic surfactant, 2.0 wt% KCl and 0.1 wt% clay stabilizer ChCl. Then, 0.5 wt% of different types of breaker agents are added to the base fluid, and the mixture is stirred at a low speed for 5 min to achieve uniform dispersion. The gel-breaking performance is tested by using an Anton Paar MCR 302 rheometer under the conditions of a temperature of 85 °C and a shear rate of 170 s^−1^, and the viscosity is measured once every hour. After the test, the sample is filtered through a 200-mesh screen. The filter residue is washed and dried, and then weighed to calculate the residue content.

## 3. Results and Discussion

### 3.1. Structural Characterization of Gel-Breaker Carriers

#### 3.1.1. FT-IR Analysis

**Figure 2:**
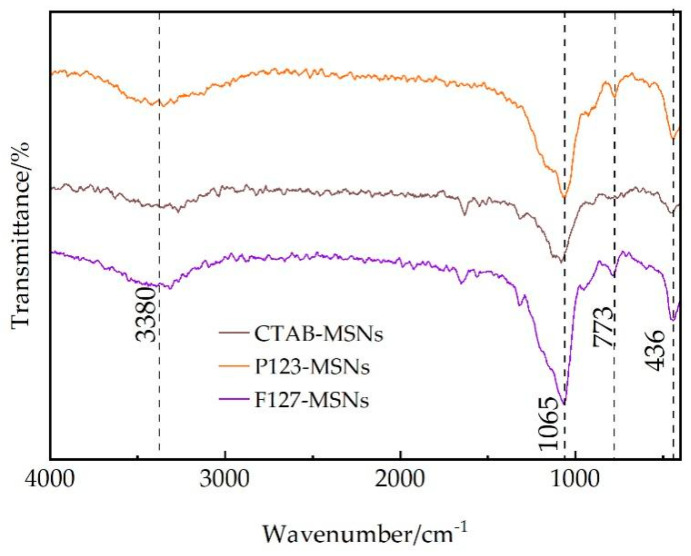
The FT-IR of CTAB-MSNs, P123-MSNs, and F127-MSNs.

Figure 2 displays the infrared spectra of three mesoporous silica materials with different pore sizes: CTAB-MSNs, P123-MSNs, and F127-MSNs. Through the FTIR spectral analysis of these three MSNs with varying pore sizes, it was observed that all samples exhibit a distinct asymmetric stretching vibration peak of Si-O-Si at 1065 cm^−1^, which is a characteristic peak of the mesoporous silica framework structure [21]. Additionally, symmetric stretching vibration peaks and bending vibration peaks of Si-O bonds can be detected at 773 cm^−1^ and 436 cm^−1^, respectively, further confirming the formation of the siloxane tetrahedral network structure [22].

Notably, the three samples show broad peaks of different intensities in the 3300–3500 cm^−1^ region, which are attributed to the O-H stretching vibrations of surface Si-OH groups and adsorbed water molecules [23]. Among them, F127-MSNs have the highest peak intensity, P123-MSNs rank intermediate, and CTAB-MSNs show the weakest intensity. This is consistent with the differences in surface silanol group density caused by different pore sizes.

The absence of the characteristic peaks of organic compounds (such as C-H stretching vibration peaks at 2800–3000 cm^−1^) in the infrared spectrum [24] indicates the complete removal of template agents. The above FTIR analysis results demonstrate that mesoporous silica nanomaterials with complete structures have been successfully prepared, and MSNs obtained under different synthesis conditions exhibit significant differences in surface chemical properties related to pore size.

#### 3.1.2. Analysis of Particle Size and Pore Size Characteristics

**Figure 3:**
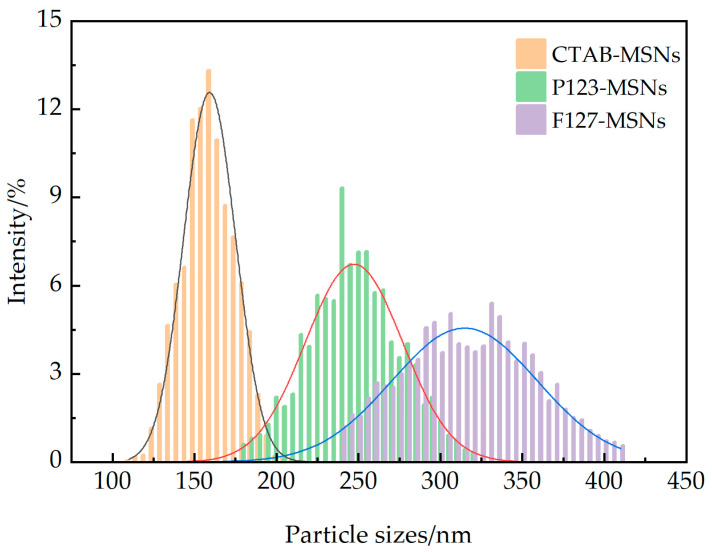
Particle size of CTAB-MSNs, P123-MSNs, and F127-MSNs.

The particle sizes and size distributions of CTAB-MSNs, P123-MSNs, and F127-MSNs nanoparticles were characterized using a nanoparticle size and zeta potential analyzer, with results shown in Figure 3 and data presented in Table A1. It can be observed that the particle sizes of the nanoparticles are relatively uniform. The measured hydrodynamic diameters (D50) of CTAB-MSNs, P123-MSNs, and F127-MSNs are 159.2 nm, 247.3 nm, and 314.8 nm, respectively, with particle size distribution ranges of 110–210 nm, 180–320 nm, and 240–410 nm, respectively. The three types of nanoparticles with different sizes all exhibit normal distribution characteristics, and their distribution intervals are relatively concentrated. The dispersity coefficients are 0.098, 0.119, and 0.141, respectively, all of which are smaller than the threshold of 0.7. This result indicates that CTAB-MSNs, P123-MSNs, and F127-MSNs all have good dispersion properties. Specifically, CTAB-MSNs show the best dispersibility, followed by P123-MSNs. Although the dispersibility of F127-MSNs is slightly worse than the former two, it still remains within the acceptable performance range.

**Figure 4:**
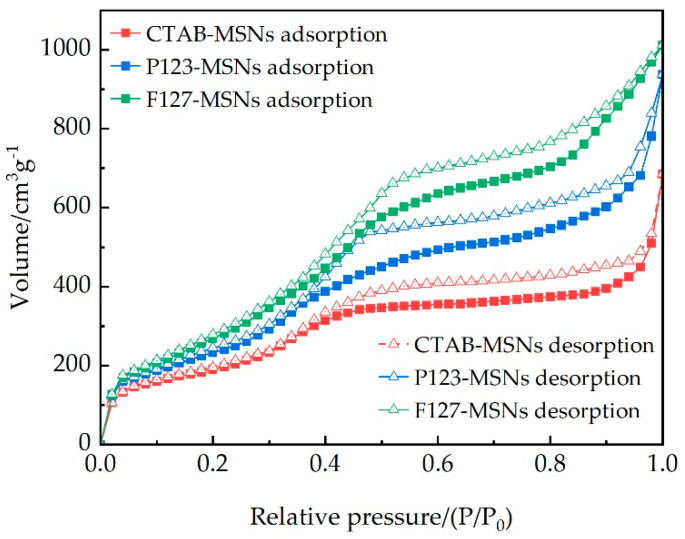
Nitrogen adsorption–desorption isotherms.

The nitrogen adsorption–desorption isotherms (Figure 4) exhibit typical Type IV curves with distinct hysteresis loops, confirming the mesoporous nature of the materials [25,26,27]. The pore structure parameters of MSNs prepared with different templating agents are listed in Table 2. As the molecular weight of the templating agent increases, the BJH pore size increases sequentially: CTAB-MSNs (5.18 nm) < P123-MSNs (6.36 nm) ≈ F127-MSNs (6.40 nm). The BET specific surface area increases from 686.08 m^2^/g to 946.89 m^2^/g, and the pore volume increases from 0.89 cm^3^/g to 1.51 cm^3^/g, representing improvements of 38.0% and 69.7%, respectively. These results indicate that larger template molecules facilitate the formation of larger pore sizes and pore volumes, while simultaneously enhancing the specific surface area.

#### 3.1.3. Thermogravimetric Curve Analysis

**Figure 5:**
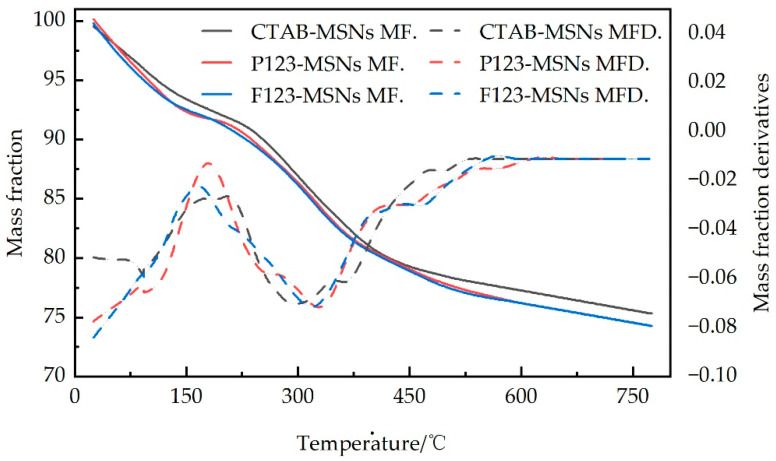
The TGA curves for different MSNs.

Figure 5 presents the thermogravimetric analysis (TGA) curves and corresponding derivative thermogravimetric (DTG) curves of CTAB-MSNs, P123-MSNs, and F127-MSNs materials under a nitrogen atmosphere within the temperature range of 25 to 750 °C. All three mesoporous silica samples exhibit typical biphasic mass loss behavior, featuring two distinct rapid degradation regions separated by a progressive weight loss stage. The initial rapid mass loss (4.0–5.2% mass loss) occurs between 25 and 100 °C, corresponding to the evaporation of physically adsorbed water, while the second accelerated degradation stage (8.6–9.4% mass loss) observed at 250–400 °C is attributed to the dehydroxylation reaction of surface silanol groups via condensation. The intermediate temperature range (100–250 °C) and the high-temperature region (400–750 °C) show relatively slow and stable mass reduction, primarily related to the gradual elimination of structurally bound water and the further densification process, respectively. DTG analysis reveals distinct decomposition peaks corresponding to these rapid mass loss events, among which CTAB-MSNs exhibit the highest thermal stability with a total mass loss of 24.0%, compared to 25.6% for P123-MSNs and 25.3% for F127-MSNs. This discrepancy is primarily attributed to the fact that CTAB, as a small-molecule surfactant, is more readily and completely removed, leading to minimal template residue and the formation of a denser and more stable siloxane framework. In contrast, macromolecular block copolymers such as P123 and F127, due to their longer molecular chains and complex structures, are more prone to residual accumulation within the pore channels, causing disturbances to the framework structure. The final residual mass ranging from 74.6% to 75.6% confirms the successful removal of organic templates during the synthesis process, indicating the effectiveness and completeness of the calcination process.

### 3.2. Characterization of Performance of Gel-Breaker Carriers

#### 3.2.1. Analysis of Loading Performance

**Figure 6:**
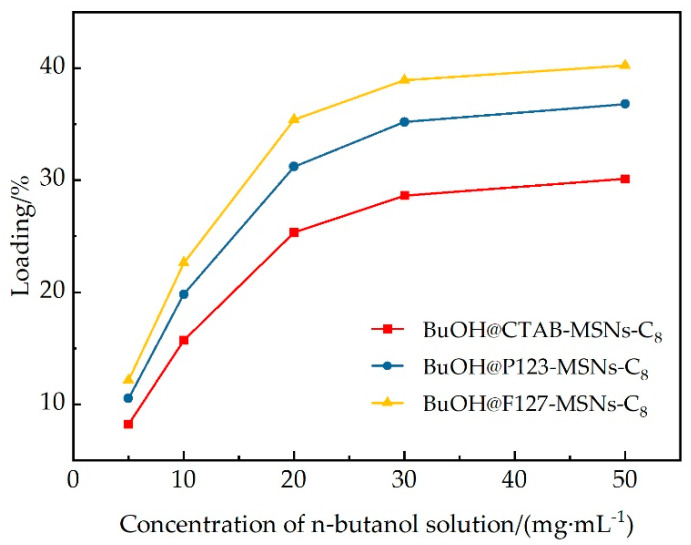
Loading ratio of BuOH@CTAB-MSNs-C_8_,P123-MSNs-C_8_, and F127-MSNs-C_8_.

Figure 6 illustrates the concentration-dependent loading behavior of n-butanol on three hydrophobized MSN (mesoporous silica nanoparticle) carriers. Experimental data indicate that the loading capacity of all carriers shows a positive correlation with the initial concentration, conforming to the typical characteristics of adsorption isotherms. At a low concentration of 5 mg/mL, the loading capacities of CTAB-MSNs-C_8_, P123-MSNs-C_8_, and F127-MSNs-C_8_ are 8.2%, 10.5%, and 12.1%, respectively, indicating preliminary performance differences among the carriers. When the concentration is increased to 30 mg/mL, the loading capacities of the three carriers significantly increase to 28.6%, 35.2%, and 38.9%, with the growth rate of loading capacity beginning to slow down. Further increasing the concentration to 50 mg/mL only slightly increases the loading capacities to 30.1%, 36.8%, and 40.2%, suggesting that the carriers are approaching a saturated loading state.

The structural basis for the performance differences among the carriers lies in the variations in their pore characteristics. F127-MSNs-C_8_ exhibits the best loading performance, which is attributed to its largest pore volume (1.51 cm^3^/g) and specific surface area (946.89 m^2^/g), providing abundant molecular accommodation space. The loading curve shows a distinct two-stage feature: the loading capacity increases sharply in the low-concentration range (5–20 mg/mL), reflecting the rapid occupation of high-energy adsorption sites; the growth tends to flatten in the high-concentration range (30–50 mg/mL), indicating the gradual exhaustion of available pore space.

Through quantitative analysis of the adsorption density under the optimal condition of 30 mg/mL, the loading capacities per specific surface area of the three carriers are found to be 0.417 mg/m^2^ for CTAB-MSNs-C_8_, 0.413 mg/m^2^ for P123-MSNs-C_8_, and 0.411 mg/m^2^ for F127-MSNs-C_8_, with highly consistent values. This result confirms the uniformity and effectiveness of OTES hydrophobization modification, indicating the consistency of n-butanol adsorption behavior on different carrier surfaces. The differences in loading capacity among carriers primarily stem from variations in pore volume rather than changes in surface chemical environment. Structure–property relationship analysis demonstrates that the pore volume advantage of F127-MSNs-C_8_ (0.62 cm^3^/g) over CTAB-MSNs-C_8_ directly correlates with a 10.3 wt% enhancement in loading capacity, quantifying the contribution of pore structure to drug-loading performance.

#### 3.2.2. Analysis of Sustained-Release Performance

**Figure 7:**
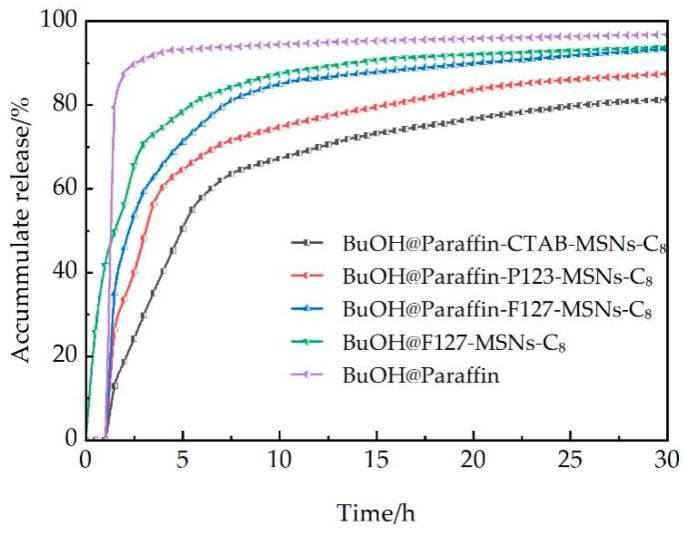
Cumulative release rates of n-butanol in different loading media.

Figure 7 illustrates the cumulative n-butanol release behaviors of different mesoporous silica nanoparticle (MSN)-supported breaker systems under simulated reservoir conditions. The delayed release effect of paraffin coating is verified through comparative analysis: the uncoated BuOH@F127-MSNs-C_8_ releases 25.86% n-butanol at 0.5 h, while the paraffin-coated system exhibits 0% release at the same time point, effectively preventing premature gel breaking during the fracturing fluid injection stage. This delayed release mechanism is highly compatible with the requirements of coalbed methane fracturing technology, where the fracturing fluid injection stage (typically 2–4 h) necessitates maintaining high viscosity to ensure effective fracture creation and proppant transportation.

**Figure 8:**
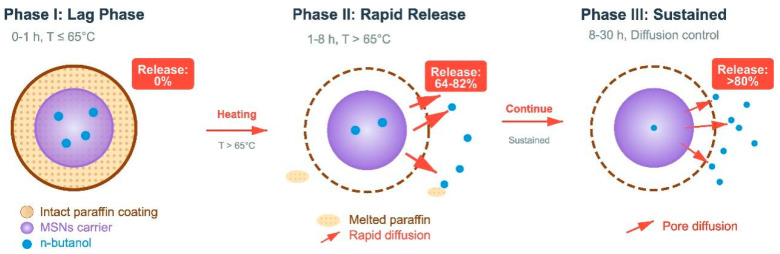
Sustained-release mechanism of paraffin-encapsulated mesoporous silica nanoparticles as a gel-breaker carrier.

As shown in Figure 8, the paraffin-encapsulated MSN system exhibits distinct three-stage release characteristics that are highly matched with the temporal requirements of coalbed methane fracturing operations. In Stage I (lag period, 0–1 h), when the reservoir temperature is below the paraffin melting point (65–70 °C), the solid paraffin encapsulation layer completely seals the carrier pores, effectively retaining n-butanol molecules with a release amount approaching 0%, ensuring the fracturing fluid maintains a high viscosity of 130–150 MPa·s to meet the requirements for effective fracture creation and proppant transport. In Stage II (rapid release period, 1–8 h), paraffin melting triggers rapid gel breaker release, with carrier pore structure significantly affecting release behavior: F127-MSNs possesses the largest pore diameter (6.40 nm) and pore volume (1.51 × 10^−6^ m^3^/g), providing spacious channels for molecular diffusion with a release increment of 82.04%; while CTAB-MSNs has smaller pore diameter (5.18 nm), resulting in increased diffusion resistance and a release increment of only 64.50%; in contrast, the burst release characteristics of unencapsulated carriers (87.38% release at 1 h) would cause rapid proppant settling and damage fracture conductivity. In Stage III (sustained release period, 8–30 h), with temperature maintained at 85 °C, the release process is primarily controlled by molecular diffusion within pores, with diffusion kinetics showing a positive correlation with carrier pore diameter: large-pore carriers exhibit higher molecular diffusion coefficients, while small-pore carriers demonstrate stronger pore confinement effects and pronounced sustained release characteristics. Considering that the strong adsorption characteristics of coal rock matrix may reduce the effective concentration of gel breakers by 20–30%, and some deep reservoirs may have temperatures below the standard paraffin melting point, optimization and adjustment according to specific reservoir conditions are required in practical applications: low-temperature reservoirs (< 65 °C) can utilize low-melting-point paraffin (50–60 °C) or paraffin mixtures, while high-adsorption coal seams require increased drug loading to 40–50 mg/mL or adoption of anti-adsorption modified carriers to ensure sufficient effective concentration of gel breaker.

The pore structure of the carrier significantly influences the release behavior. The cumulative release amounts at the 8-h time point follow the order of F127-MSNs-C_8_ > P123-MSNs-C_8_ > CTAB-MSNs-C_8_, which is directly correlated with the carrier’s pore size and pore volume parameters. The rapid release phase (1–8 h) represents a critical stage for the breaker to exert its function. The release increments of the three systems are 82.04%, 72.15%, and 64.50%, respectively, enabling them to reach the critical concentration required for VES fracturing fluid gel breaking within a short period. Although the BuOH@Paraffin control system also exhibits a delayed effect, it releases 87.38% at 1 h, it has an adverse impact on the stable placement of proppants within fractures.

From the perspective of practical applications in coalbed methane reservoirs, the cumulative release amounts of the three carrier systems within the 6–12 h gel-breaking window are 57.85–69.82% (CTAB), 67.73–76.78% (P123), and 75.34–86.41% (F127), respectively, all exceeding the minimum requirements for effective gel breaking. The final cumulative release data at 30 h (CTAB: 81.25%, P123: 87.39%, F127: 93.26%) indicate that the carriers can achieve nearly complete release of the breaker, verifying the stability of the drug-loading method and mesoporous network structure. However, the strong adsorption properties of coal rocks may reduce the effective concentration of the breaker and, therefore, the temperature of some coalbed methane reservoirs may be lower than the melting point of paraffin (65–70 °C); the optimization and adjustment of the paraffin composition and drug-loading amount according to the specific reservoir conditions is required in practical applications. Comprehensive evaluation of release efficiency, time control, and engineering adaptability reveals that the F127-MSNs-C_8_ system exhibits the optimal application potential.

### 3.3. Comparative Analysis of Gel Breaking Degree

#### 3.3.1. Analysis of Viscosity–Time Curve

**Figure 9:**
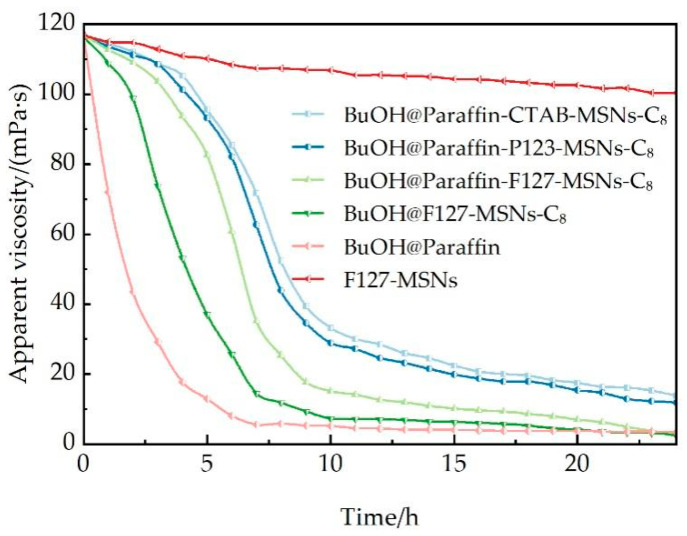
Apparent viscosity–time relationship curves of different gel breakers.

As illustrated in Figure 9, the paraffin-encapsulated MSN system exhibits remarkable temperature-controlled delayed gel-breaking characteristics, demonstrating a three-stage release behavior under reservoir conditions at 85 °C. During the initial induction stage (0–3 h), all encapsulated systems maintain a high-viscosity state (103–117 MPa·s) with a viscosity retention rate > 88%, effectively preventing premature gel breaking during the fracturing injection stage. When the reservoir temperature exceeds the paraffin melting point (65–70 °C), the rapid gel breaking stage (3–8 h) begins, with paraffin undergoing phase transition from a solid to liquid state. The encapsulation layer structure completely fails, reopening the sealed carrier pores and promoting rapid diffusion of n-butanol molecules into the fracturing fluid, disrupting the intermolecular hydrogen bonding network of VES molecules, causing wormlike micelles to transform into spherical micelles, and resulting in viscosity dropping sharply from 103–117 MPa·s to 15–35 MPa·s. The subsequent sustained release stage (8–24 h) achieves continuous release through the porous structure of the mesoporous carrier, ensuring the integrity of the gel breaking process. Control experiments show that the unencapsulated carrier BuOH@F127-MSNs-C_8_ had its viscosity reduced to 73.8 MPa·s at 3 h, while the paraffin-encapsulated system still maintained >103 MPa·s, confirming the temperature-controlled delay effect of paraffin encapsulation. The blank carrier F127-MSNs showed viscosity decreasing only from 116.8 MPa·s to 100.3 MPa·s within 24 h (viscosity retention rate of 85.9%), indicating that silica carriers, as inert materials, exhibit only weak physical adsorption interactions between surface silanol groups and VES molecules, insufficient to disrupt micellar structure, and thereby validating that the carrier material itself does not possess gel breaking capability.

Based on the BJH pore size distribution analysis results, the pore size range of mesoporous carriers is concentrated in the 4–8 nm interval, with CTAB-MSNs mainly distributed in 4.5–5.8 nm, P123-MSNs in 5.8–7.1 nm, and F127-MSNs in 5.9–7.2 nm. The pore size distribution directly affects the release kinetics and final gel breaking degree of the n-butanol gel breaker. The BuOH@Paraffin-F127-MSNs-C_8_ system exhibits optimal gel breaking performance, with a 24 h gel breaking degree of 97.5% (final viscosity 2.91 MPa·s), significantly superior to the P123 carrier’s 89.9% (11.79 MPa·s) and CTAB carrier’s 88.1% (13.78 MPa·s). According to porous media diffusion theory, larger pore sizes favor reducing the diffusion resistance of n-butanol molecules, promoting the mass transfer process of molecules within carrier pores, enabling the gel breaker to reach critical destructive concentration (approximately 15–20 mM) in VES micelles, thoroughly disrupting intermolecular hydrogen bonding forces, and achieving complete transformation from wormlike micelles to spherical micelles. The gel breaking degree data at the 8 h critical time point (F127: 78.3%, P123: 62.5%, CTAB: 55.0%) shows a positive correlation with carrier pore volume parameters (1.51, 1.36, 0.89 cm^3^/g), indicating that increased pore volume contributes to enhanced gel breaking degree, confirming the decisive role of pore structure parameters on gel breaking performance.

The developed paraffin-encapsulated MSN gel breaker system is highly matched with the temporal sequence of coalbed methane fracturing operations. During the injection and fracturing stage (0–3 h), the encapsulated system effectively maintains high viscosity > 103 MPa·s, meeting the requirements for sand carrying and fracture creation; during the shut-in stage (3–6 h), reservoir temperature activates gel breaker release, with F127 system viscosity moderately decreasing to 60.7 MPa·s; during the flowback and production enhancement stage (6–24 h), viscosity further decreases to low values of 2.9–13.8 MPa·s, maximizing the reduction of flowback resistance.

#### 3.3.2. Analysis of Residue Content

As is shown in Table 3, residue content analysis reveals that at 0.3 wt% dosage, the total residue contents of F127-, P123-, and CTAB-encapsulated systems are 46.0, 56.3, and 60.2 mg/L, respectively. The lowest residue content of BuOH@Paraffin-F127-MSNs-C_8_ is attributed to three key factors: enhanced particle dispersion due to the largest specific surface area (946.89 m^2^/g) and pore volume (1.51 × 10^−6^ m^3^/g), which minimizes aggregation-induced large particle residue formation; complete gel breaker release (93.26%) is facilitated by larger pore diameter (6.40 nm), and thereby reduces organic residue from the retained gel breaker within carriers; and more thorough template removal under lower calcination temperature (350 °C), resulting in reduced organic residue in the carrier framework.

The superior gel breaking efficiency (97.5%) of the F127-MSNs-C_8_ system is substantiated by three aspects: optimal drug-loading capacity (38.9%) ensuring sufficient gel breaker dosage; rapid release increment of 82.04% during the three-stage release mode guaranteeing effective concentration within the critical time window; and uniform pore structure promoting homogeneous distribution and complete action of the gel breaker. The residue primarily consists of amorphous SiO_2_ nanoparticles and trace organic compounds, demonstrating excellent environmental compatibility.

Based on comprehensive evaluation of gel breaking degree, process compatibility, and reservoir protection effectiveness, BuOH@Paraffin-F127-MSNs-C_8_ represents the optimal formulation for coalbed methane fracturing operations in reservoirs with temperatures > 70 °C.

### 3.4. Comprehensive Assessment and Future Perspectives

The proposed mesoporous silica-based gel breaker system demonstrates significant potential for industrial implementation. The synthesis method utilizes commercially available precursors (TEOS, CTAB, P123, F127) and well-established sol-gel processes, and it operates under moderate conditions (60–100 °C) with standard equipment requirements, indicating favorable scalability prospects. From an environmental standpoint, the synthesis process exhibits good green chemistry characteristics: water and ethanol serve as primary solvents, hexane used in paraffin coating can be recovered through distillation, and the final SiO_2_-based carriers are inherently biodegradable with minimal environmental impact.

This study provides a comprehensive performance evaluation beyond conventional gel breaking efficiency metrics. Key performance indicators include controlled release kinetics across three distinct phases, residue content as low as 46.0 mg/L, exceptional thermal stability (up to 750 °C), optimal particle dispersibility, and loading efficiencies reaching 38.9%. The systematic structure–property relationships established between carrier pore parameters and performance outcomes offer valuable insights for system optimization.

Despite these promising results, several limitations warrant consideration for practical implementation: (1) pilot-scale validation is essential to confirm the scalability from laboratory synthesis to industrial production; (2) long-term stability and performance consistency under diverse reservoir conditions (varying temperature, pressure, and formation water composition) require extensive field testing; and (3) comprehensive economic analysis including a cost–benefit evaluation and a market competitiveness evaluation needs to be conducted. Future research directions should prioritize pilot-scale field trials, development of alternative triggering mechanisms for lower-temperature reservoirs, investigation of carrier recyclability and environmental fate, and optimization of synthesis parameters for commercial viability. Additionally, expanding the applicability to other unconventional gas reservoirs and integration with existing fracturing technologies represent important avenues for further development.

## 4. Conclusions

This study successfully constructed an intelligent controlled-release gel breaker system based on paraffin-encapsulated mesoporous silica nanocarriers and systematically evaluated its application performance in coalbed methane fracturing fluids. The main conclusions are as follows:

(1) Using the sol-gel method with surfactant (CTAB) and block copolymers (P123, F127) of different molecular sizes as structure-directing agents, mesoporous silica carriers with tunable pore sizes were successfully prepared. With increasing template molecule size, carrier pore diameter increased from 5.18 nm to 6.40 nm, while specific surface area and pore volume increased by 38.0% and 69.7%, respectively. F127-MSNs-C_8_ achieved a drug-loading efficiency of 38.9%, with carrier pore volume being the primary factor affecting loading performance.

(2) The paraffin encapsulation technique achieved the temperature-responsive controlled release of gel breakers, forming a three-stage release pattern highly matched with coalbed methane fracturing operations: lag period (0–1 h) with release amount ≈0% ensuring viscosity maintenance during injection and fracturing stage; rapid release period (1–8 h) with release increment of 64.5–82.0% meeting timely gel breaking requirements; sustained release period (8–30 h) with continuous gel breaker release, achieving effective matching between gel breaking timing and fracturing processes.

(3) BuOH@Paraffin-F127-MSNs-C_8_ system exhibited optimal performance: 24 h gel breaking degree reached 97.5% (final viscosity 2.91 MPa·s), significantly superior to P123- and CTAB-based carriers. At 0.3 wt% dosage, total residue content was only 46.0 mg/L, meeting environmental requirements.

(4) The developed intelligent gel breaker demonstrates excellent temporal coordination with coalbed methane fracturing operations, maintaining high viscosity (>103 MPa·s) during injection stage to ensure sand-carrying capacity, and rapidly decreasing to low viscosity (<15 MPa·s) during the flowback stage to reduce flow resistance. BuOH@Paraffin-F127-MSNs-C_8_ represents the optimal formulation for coalbed methane fracturing in reservoirs with temperatures above 70 °C.

## Figures and Tables

**Table 1:** Experimental instruments.

Name	Model Number	Supplier
Vacuum Freeze–Dryer	FD-1B-50	Beijing Boyikang Exp. Ins. Co., Ltd. (Beijing, China)
Electronic Analytical Balance	BSA224S	Germany Sartorius Scientific Instruments Company (Göttingen, Germany)
Flat-Type Viscometer	B-013102	Shanghai Changji Geological Instrument Co., Ltd. (Shanghai, China)
Magnetic Stirrer	IKA RCT basic	The IKA Company of Germany (Staufen im Breisgau, Germany)
Muffle Furnace	SX2-4-10	Shanghai Yuejin Medical Device Co., Ltd. (Shanghai, China)
High–Speed Centrifuge	TGL-16M	Shanghai Anting Scientific Instrument Factory (Shanghai, China)
Vacuum Drying Oven	DZF-6020	Shanghai Yiheng Scientific Instruments Co., Ltd. (Shanghai, China)
High-Pressure Hydrothermal Reactor	KH-50 mL	Beijing Kewei Yongxing Instrument Co., Ltd. (Beijing, China)
Six–Speed Rotational Viscometer	ZNN-D6B	Shanghai KenCe Instruments Co., Ltd. (Shanghai, China)
Zeta Potential Analyzer	Zetasizer ZS90	Malvern Instruments Limited (Worcestershire, UK)

**Table 2:** Specific surface area and pore volume data of mesoporous silica nanoparticles (MSNs) with different pore sizes.

Sample	Diameter of Hole (nm)	Specific Surface Area (m^2^/g)	Pore Volume (m^3^/g)
CTAB-MSNs	5.18	686.08	8.9 × 10^−7^
P123-MSNs	6.36	853.17	1.36 × 10^−6^
F127-MSNs	6.40	946.89	1.51 × 10^−6^

**Table 3:** Analysis of residue content.

System	Gel Breaking Efficiency (%)	Total Residue (mg/L)
BuOH@Paraffin—F127-MSNs-C_8_	97.5	46.0
BuOH@Paraffin—P123-MSNs-C_8_	89.9	56.3
BuOH@Paraffin—CTAB-MSNs-C_8_	88.1	60.2

## Data Availability

The original contributions presented in this study are included in the article material. Further inquiries can be directed to the corresponding author(s).

## Appendix A

**Table A1:** Particle size of CTAB-MSNs, P123-MSNs, and F127-MSNs.

Particle Size/nm	Intensity of CTAB-MSNs/%	Particle Size/nm	Intensity of P123-MSNs/%	Particle Size/nm	Intensity of F127-MSNs/%
110–115	0.04	180–185	0.60	240–245	1.20
115–120	0.16	185–190	0.81	245–250	1.62
120–125	0.24	190–195	0.93	250–255	1.56
125–130	1.14	195–200	1.29	255–260	2.17
130–135	2.65	200–205	2.20	260–265	2.70
135–140	4.65	205–210	1.89	265–270	2.57
140–145	6.05	210–215	2.31	270–275	2.54
145–150	6.63	215–220	4.34	275–280	2.98
150–155	11.65	220–225	3.93	280–285	3.43
155–160	12.04	225–230	5.67	285–290	3.49
160–165	13.32	230–235	5.55	290–295	4.57
165–170	10.96	235–240	5.50	295–300	4.75
170–175	8.73	240–245	9.33	300–305	3.72
175–180	7.65	245–250	6.72	305–310	5.06
180–185	6.10	250–255	7.15	310–315	4.02
185–190	4.43	255–260	7.16	315–320	3.92
190–195	2.30	260–265	5.77	320–325	3.75
195–200	0.89	265–270	5.85	325–330	3.96
200–205	0.20	270–275	4.07	330–335	5.41
205–210	0.14	275–280	3.55	335–340	4.96
210–215	0.03	280–285	4.02	340–345	4.08
		285–290	3.32	345–350	3.46
		290–295	1.95	350–355	4.03
		295–300	2.21	355–360	3.65
		300–305	1.41	360–365	3.04
		305–310	0.93	365–370	2.09
		310–315	0.77	370–375	2.65
		315–320	0.43	375–380	1.80
		320–325	0.33	380–385	1.51
				385–390	1.43
				390–395	1.07
				395–400	0.90
				400–405	0.72
				405–410	0.67
				410–415	0.56
